# Peramivir, an Anti-Influenza Virus Drug, Exhibits Potential Anti-Cytokine Storm Effects

**DOI:** 10.3389/fimmu.2022.856327

**Published:** 2022-02-28

**Authors:** Chen-xi Zhang, Ye Tu, Xiao-chen Sun, Da-gui Chen, Wan-nian Zhang, Chun-lin Zhuang, Zhi-bin Wang, Li Su

**Affiliations:** ^1^ Institute of Translational Medicine, Shanghai University, Shanghai, China; ^2^ Department of Pharmacy, Shanghai East Hospital, Tongji University, Shanghai, China; ^3^ School of Medicine, Shanghai University, Shanghai, China; ^4^ School of Pharmacy, Naval Medical University, Shanghai, China; ^5^ School of Pharmacy, Ningxia Medical University, Yinchuan, China; ^6^ Department of Critical Care Medicine, School of Anesthesiology, Naval Medical University, Shanghai, China

**Keywords:** cytokine storm syndrome, COVID-19, peramivir, acute lung injury, multi-cytokines

## Abstract

Coronavirus Disease 2019 (COVID-19) infected by Severe Acute Respiratory Syndrome Coronavirus-2 (SARS-CoV-2) has been declared a public health emergency of international concerns. Cytokine storm syndrome (CSS) is a critical clinical symptom of severe COVID-19 patients, and the macrophage is recognized as the direct host cell of SARS-CoV-2 and potential drivers of CSS. In the present study, peramivir was identified to reduce TNF-α by partly intervention of NF-κB activity in LPS-induced macrophage model. *In vivo*, peramivir reduced the multi-cytokines in serum and bronchoalveolar lavage fluid (BALF), alleviated the acute lung injury and prolonged the survival time in mice. In human peripheral blood mononuclear cells (hPBMCs), peramivir could also inhibit the release of TNF-α. Collectively, we proposed that peramivir might be a candidate for the treatment of COVID-19 and other infections related CSS.

**Graphical Abstract:**
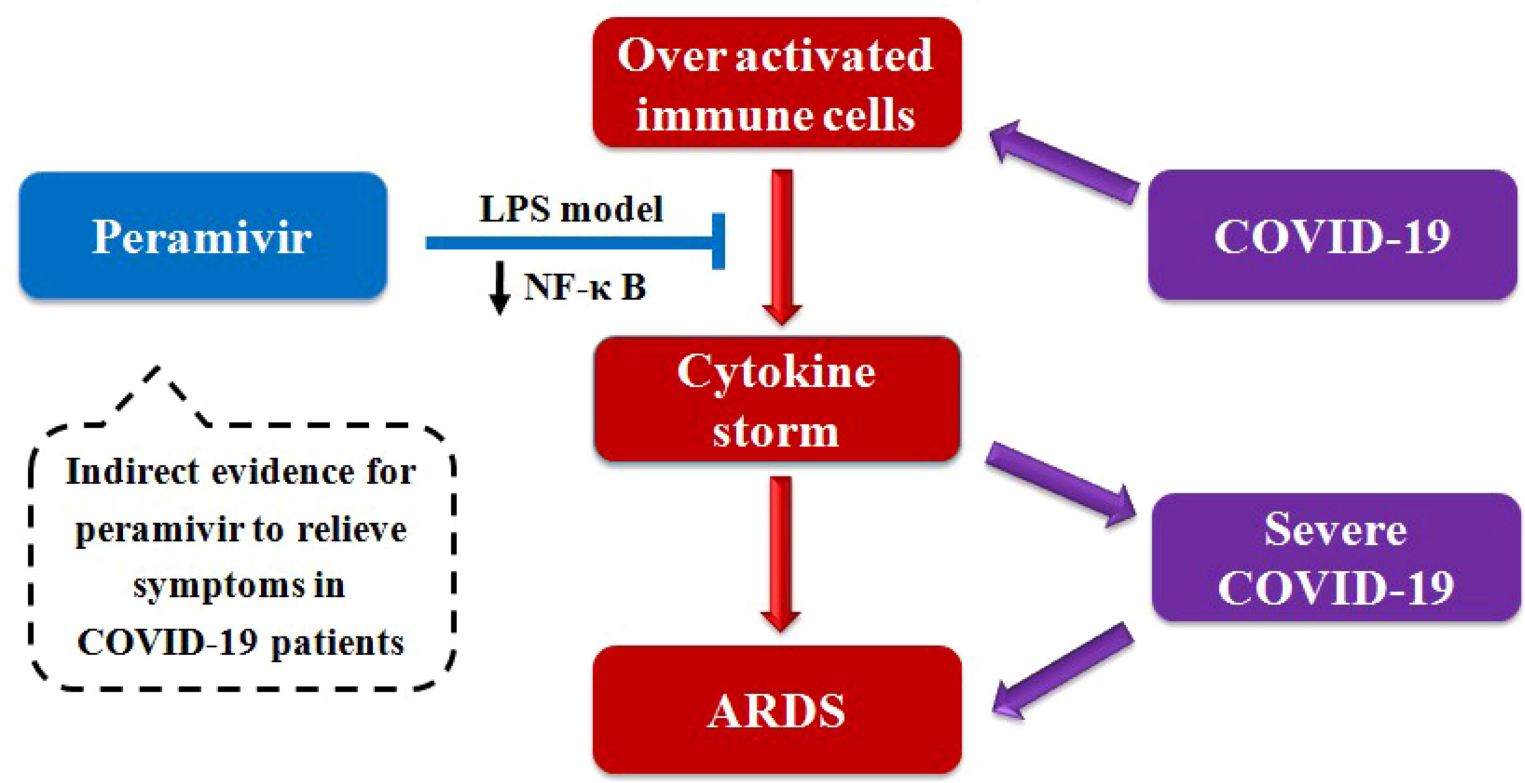


## Introduction

Coronavirus disease 2019 (COVID-19) caused by Severe Acute Respiratory Syndrome Coronavirus -2 (SARS-CoV-2) has been reported that more than 231.5 million people were infected and 4.85 million people of them were killed. (updated on Nov 9^th^, 2021) (1). World Health Organization (WHO) has declared that information sharing, strategic corporation and efficient management of medical resources are critical for global public health (2). COVID-19 patients show typical clinical symptoms of fever, fatigue, dry cough and pneumonia (3–5). Excessive inflammatory response leads to acute respiratory distress syndrome (ARDS), coagulopathy, and septic shock which can be fatal in critical cases (3). Gross anatomy identifies the main pathological features including exudation and hemorrhage, epithelium injuries, infiltration of inflammatory immune cells and fibrosis in the lungs of fatal patients (3, 6–10).

A syndrome with a distinct cytokine storm was showed in a subgroup of patients with severe COVID-19, which has also been reported in SARS-CoV infected patients (11–14). The cytokine storm refers to an uncontrolled excessive inflammatory response, spreading from a local lesion to the whole body through the systemic circulation (15, 16). The inflammation related cytokines such as interleukins (IL) -2, IL-6, IL-7, and IL-10, tumor necrosis factor-α (TNF-α), interferon-γ-inducible protein 10 (IP10), granulocyte-colony stimulating factor (G-CSF), monocyte chemoattractant protein -1 (MCP-1), and macrophage inflammatory protein 1 alpha (MIP-1α) in the plasma of COVID-19 patients were significantly increased (3, 17, 18). Specially, the activation of alveolar macrophages was a characteristic abnormality (8).

The leading cause of mortality is thought to be ARDS-induced respiratory failure, and patients generally receive supportive management in clinic practice (17). However, there is still no well-accepted effective treatment for COVID-19. The current therapeutics are mainly focused on antivirals (19, 20) and vaccines (11). In addition, the cytokine storm is increasingly being recognized as a key node for the patients deteriorating to severe COVID-19 (3, 11, 17). Therefore, anti-inflammatory therapy has been considered as one of appropriate clinical adjuvant treatment options, and the treatments include steroids (e.g., prednisone) (21), selective cytokine blockade (e.g., tocilizumab) (11), JAK inhibition (e.g., Baricitinib) (22), intravenous immunoglobulin, Chinese medicines and blood purification (11, 21).

Early in Feb, 2020, 75 of 99 COVID-19 patients received antiviral treatment including oseltamivir (23). Besides, oseltamivir is noted to have been widely used for confirmed or suspected COVID-19 cases in hospitals in China (24) and Thailand (NCT04303299). The other two clinical trials (NCT04261270 and NCT04255017) involving oseltamivir in the treatment of COVID-19 are currently ongoing. However, the FDA-approved neuraminidase inhibitors including oseltamivir, zanamivir and peramivir were ineffective against the SARS-CoV-2 virus *in vitro* (25). There has been no exact evidence to date that oseltamivir is effective in the treatment of COVID-19 in clinic (19).

It is reported that oseltamivir exhibited the antiviral activity of reducing pulmonary viral load, thereby the cytokines production was suppressed (26). Macrophages play a vital role in both SARS-CoV-2 virus -induced lung lesions and the host cytokine-mediated response (8). In our previous study, entecavir, a hepatitis B virus (HBV) inhibitor, was demonstrated to directly inhibit the release of cytokines in lipopolysaccharide (LPS) -stimulated macrophage model (27), which is a classic *in vitro* model to evaluate the anti-inflammatory activity of the drugs (28). Herein, we also examined whether these three neuraminidase inhibitors could inhibit the expression of inflammatory cytokines in LPS-stimulated macrophages in the present study. The results showed peramivir had the best ability to inhibit TNF-α by ~70% among the three compounds. Furthermore, we estimated the effect of anti-cytokine storm and lung protection of peramivir *in vivo*. The anti-inflammatory effect of peramivir on human peripheral blood mononuclear cells (hPBMCs) was also observed.

## Materials and Methods

### Materials

Compounds were purchased from TargetMol with a purity of > 98% (TargetMol). LPS (*E*. *coli* 0111:B4) was obtained from MilliporeSigma.

### Animal

Thirty 8-week-old male C57BL/6J mice (purchased from the Changzhou Cavens Laboratory Animal Co, China) were prepared. All mice had free access to standard dry food and purified water. Mice were grown in a temperature-controlled environment at 22°C ± 2°C with 12 light/dark cycles and 50%– 60% relative humidity. All animal experiments were carried out in adherence with the NIH Guide for the Care and Use of Laboratory Animals (National Academies Press, 2011) and were approved by the Second Military Medical University Committee on Animal Care (EC11-055).

### Cytokine Storm Syndrome model

CCS mouse model was established by a single intraperitoneal (i.p.) injection of LPS (15 mg/kg) as described previously (29). Mice were sacrificed 4 hours after LPS injection and serum was collected. Ligated the left bronchus and perfused 1 mL saline into right lung lobe to collect BALF after 8 h, the right lung was perfused with 1 mL saline, and the BALF was collected and the left lung was fixed with 4% paraformaldehyde for histological analysis. Serum and BALF were further used for multi-cytokine analysis. Next, we used a single i.p. injection of saline and peramivir to investigate the effect of the drugs on the survival time of the CSS model. Mice were randomly divided into three groups (n=10 per group): saline group, low-dose peramivir (20 mg/kg), and high-dose peramivir (60 mg/kg). Saline and peramivir were administered 1 hour before i.p. injection. Mice were injected with a lethal dose of LPS (30 mg/kg). The survival time of mice was recorded every 2 hours until 40 hours.

### Preparation of the Peritoneal Macrophages

I.p. injection 3 ml of 3% thioglycolate to induce peritoneal macrophages as described previously (29). Mice were sacrificed and peritoneal cavity was lavaged with 5 ml of RPMI 1640 (Gibco Life Technologies) to collect macrophages. After attachment, the cells were washed twice with PBS, added to RPMI 1640 medium and incubated at 37°C in a 5% CO2 incubator. Finally, the cells were stimulated with 100 ng/ml LPS to collect the supernatant. Isolated cells were used for cytokine analysis and cell viability assay.

### Preparation of the hPBMCs

hPBMCs were obtained from freshly collected buffy coat fractions from healthy donors at the Tongren Hospital Affiliated to Shanghai Jiaotong University, China. Briefly, hPBMCs were isolated by centrifugation (Ficoll-Paque, Pharmacia, Uppsala, Sweden) density gradient in a Sorvall RT6000B (800 g, RT, 20 min, DuPont, Wilmington, DE, USA). The majority of hPBMC isolates were adherent cells, mainly including macrophages and monocytes. Isolated hPBMCs were cultured in RPMI 1640 with 100 U/mL penicillin-streptomycin (Invitrogen Life Technologies) and 10% heat-inactivated fetal bovine serum (Gibco Life Technologies). Cells were seeded in 96-well plates at a density of 3×10^5^ cells/well and cultured in a humidified atmosphere of 95% air and 5% CO2 for 24 hours at 37°C in an incubator. hPBMCs were pretreated with different concentrations of peramivir (2.5, 5, and 10 μM) for 1 hour, then stimulated with LPS (100 ng/ml), and supernatants were collected for cytokine analysis 6 or 12 hours after LPS stimulation.

### Cell Viability Assay

Cell Counting Kit-8 (TY0312, Dojindo Molecular Technology, Japan) was used to examine cell viability. Cells were seeded in 96-well plates, and 10μL of CCK-8 solution was added to each well after the cells were adherent, and the cells were incubated at 37°C for 1 hour. Absorbance was measured at a wavelength of 450 nm by using a Cytation 5 Cell Imaging Multi-mode Reader (BioTek Instruments, USA).

### Anti-Inflammatory Activity Screening

TNF-α was induced by LPS-stimulated peritoneal macrophages *in vitro*. Potential anti-inflammatory molecules were screened from antiviral and antibacterial drug libraries. Briefly, peritoneal macrophages were stimulated with 100 ng/ml LPS for 4 hours at a compound concentration of 10 μM. Cell supernatants were diluted 10-fold, and the concentration of TNF-α was measured using the mouse TNF-α Elisa kit from Invitrogen. The remaining cells were examined for cytotoxicity by the CCK8 assay.

### NF-κB Luciferase Activity Assay

RAW264.7 stably transfected with an NF-κB-responsive luciferase construct, kindly provided by Prof. An Qin (Shanghai Jiaotong University, China), were seeded in 96-well plates at a density of 2 × 10^5^ cells/well and allowed to incubate for 24 h (30). Cells were pretreated with peramivir for 1 h followed by stimulation with 100 ng/ml LPS for 6 h. Cells were collected and luciferase activity was measured by using a luciferase assay system (Promega) and a Cytation 5 Cell Imaging Multi-mode Reader (BioTek Instruments, USA).

### Western Blotting

Protein samples were separated by 10% sodium dodecyl sulfate-polyacrylamide gel electrophoresis (SDS-PAGE), transferred to NC membranes, and blocked with TBST of 5% skim milk for 1 hour. The membranes were washed with TBST and then incubated with the specific primary antibody for 6 hours at 4°C. The membranes were then incubated for 1 hour at room temperature in secondary and the signal was detected by chemiluminescence (Bio rad, USA). The primary (1:1000) and secondary (1:10000) antibodies were purchased from Cell Signaling Technology, USA and listed as follows: GAPDH antibody(#2118), stat3 antibody(#12640), phospho-stat3 antibody(#98543), SAPK/JNK antibody(#9252), phospho-SAPK/JNK antibody(#4668), p65 antibody(#4764), phospho-p65 antibody(#3033), IKKα antibody(#2682), phospho-IKKα/β antibody(#2697), IκBα antibody(#4812), phospho-IκBα antibody(#2859), p38 MAPK antibody(#8690), phospho-p38 MAPK antibody(#4511), Erk1/2 antibody(#4695), phospho-Erk1/2 antibody(#4370) and HRP-linked goat anti-rabbit IgG(#7074).

### ELISA

TNF-α released by mouse peritoneal macrophages and hPBMCs were measured by Mouse TNF-α ELISA Kit (Invitrogen, BMS607-3TEN) and Human TNF-α ELISA Kit (Youda, 1117202) according to the manufacturer’s protocol.

### Multi-Cytokine Measurement

The level of total 12 virus-related cytokines in serum were measured by a bead-based immunoassay panel (Mouse Anti-Virus Panel, Cat No: 740622, Biolegend, USA). The level of total 12 inflammatory cytokines in BALF were measured by a bead-based immunoassay panel (Mouse Inflammation Panel, Cat No: 740446, Biolegend, USA) on CytoFLEX Flow Cytometer (Beckman Coulter, USA) according to the manufacturer’s protocol.

### Histological Analysis

The left lung lobe of mice was fixed with formalin and embedded with paraffin. Formalin-fixed tissue sections (5 μm) were stained with hematoxylin and eosin (H&E) according to the manufacturer’s instructions and photographed with a microscope (Olympus Corporation, Tokyo, Japan). Histological features of lung injury (including alveolar edema and hemorrhage, number of infiltrating leukocytes, thickness of alveolar walls and epithelium) were assessed. Each histological feature was assessed on a scale of 0 to 3 (0, normal; 1, mild; 2, moderate; 3, severe).

### Statistical Analysis

Data were expressed as means ± SEM. Statistical analyses used Student’s *t*-test, two-way ANOVA or Kaplan-Meier Survival Analysis. GraphPad software was used for data analysis. Statistical significance was indicated as follows: *P < 0.05, **P < 0.01, ***P < 0.001, n.s. not significant.

## Results

### Peramivir Is an Active Anti-Inflammatory Agent Without Apparent Cytotoxicity

**Figure 1:**
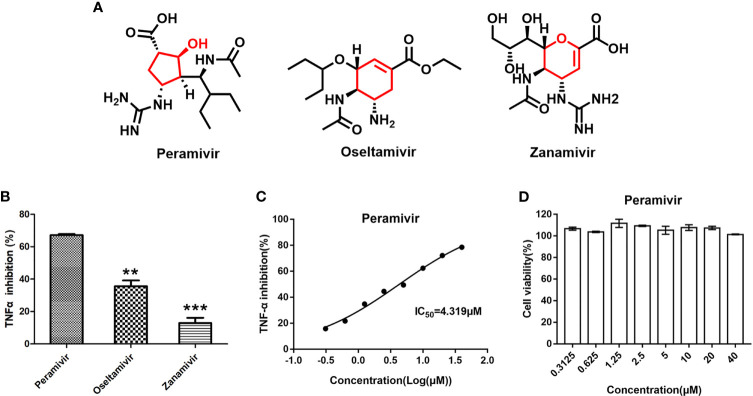
Identification of peramivir as anti-inflammatory agents. **(A)** Chemical structures of peramivir, oseltamivir and zanamivir. **(B)** Peramivir showed the strongest TNF-α inhibitory effect compared with oseltamivir and zanamivir, **P < 0.01, ***P < 0.001 vs peramivir. **(C)** The dose-response curves for the TNF-α inhibitions of peramivir exhibited IC_50_s of 4.3 µM. **(D)** Cell viabilities of macrophages with peramivir treatment at different concentrations. N = 3.

The three neuraminidase inhibitors (peramivir, oseltamivir, and zanamivir, **Figure 1A**) were explored for their ability to inhibit TNF-α-induced by LPS in macrophages. They inhibited the elevation of TNF-α by 67.2%, 35.6% and 13.1% at 10 µM, respectively (**Figure 1B**). Furthermore, peramivir dose-dependently inhibited TNF-α release with the half-maximal inhibitory concentration (IC_50_) as 4.3 µM (**Figure 1C**). We tested the cytotoxicity of peramivir in macrophages by a CCK-8 assay to eliminate the inhibitory effect on TNF-α might be achieved by cytotoxicity. Results demonstrated that no apparent toxicity was observed in the peramivir-treated macrophages at concentrations up to 40 μM (**Figure 1D**).

### Peramivir Inhibited LPS-Induced Cytokine Storm in Mice

**Figure 2:**
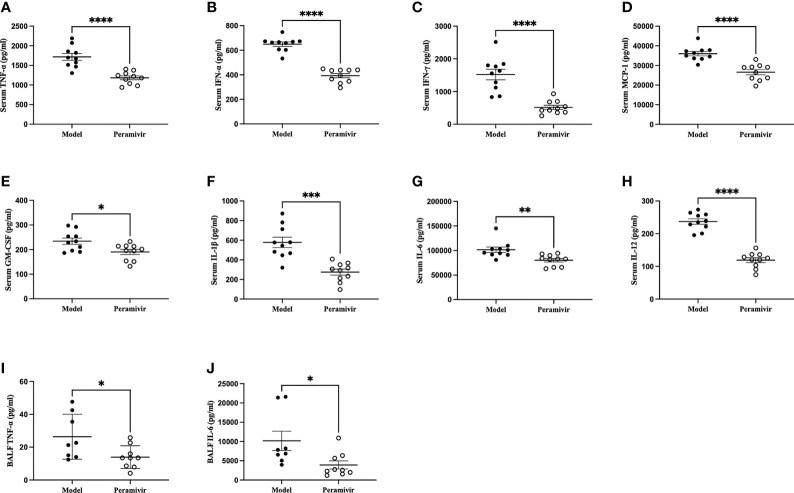
Peramivir has a significant effect on some cytokines in mouse serum and bronchoalveolar lavage fluid (BALF). **(A–H)** Serum cytokines. **(I, J)** BALF cytokines. *P < 0.05, **P <0.01, ***P < 0.001, ****P < 0.0001. N = 8-10.

**Figure 3:**
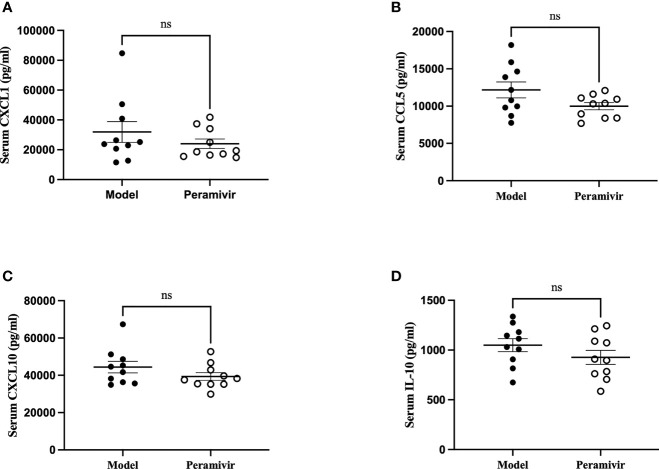
The weak effect of peramivir on chemokines and IL -10 in the serum of mice. **(A–C)** serum chemokines: CXCL1, CCL5, CXCL10. **(D)** serum cytokines: IL-10. ns, no significance. N = 10.

**Figure 4:**
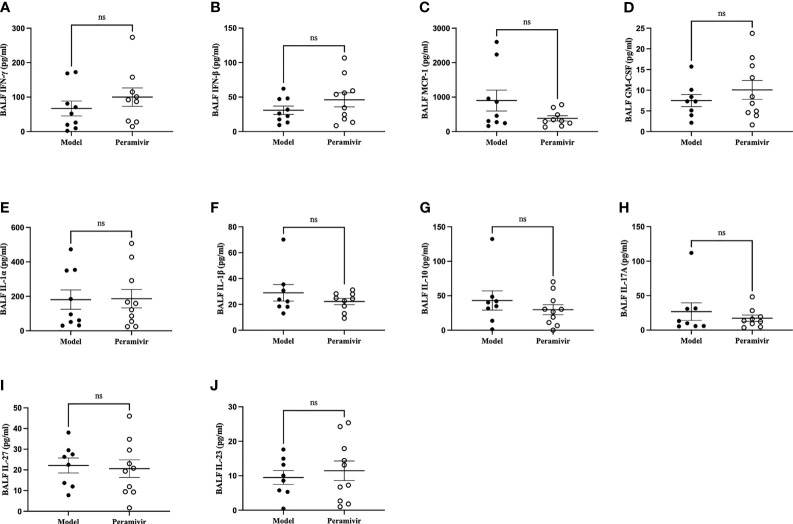
The weak effect of peramivir on some cytokines in the bronchoalveolar lavage fluid (BALF) of mice. **(A, B)** BALF cytokines: IFN-γ, IFN-β. **(C)** BALF chemokines: MCP-1. **(D)** BALF cytokines: GM-CSF. **(E–J)** BALF cytokines: IL-1α, IL-1β, IL-10, IL-17A, IL-27, IL-23. ns, no significance. N = 8-10.

*In vivo* inflammatory inhibitory activity was measured by using LPS-induced CSS mouse model. Peramivir (60 mg/kg) was administrated intraperitoneally 1 h before LPS injection, and serum was collected at 4 h after LPS injection for further experiment. Twelve cytokines in total were measured using a mouse antivirus panel by flow cytometric bead array at the same time. Results show that 8 cytokines including TNF-α, IFN-α, IFN-γ, chemokines (MCP-1), GM-CSF, IL-1β, IL-6 and IL-12 were significantly decreased by the treatment of peramivir compared with the model group (**Table 1** and **Figures 2A–H**). The other three chemokines including CXC chemokine ligand 1 (CXCL1), chemokine (C-C motif) ligand 5 (CCL-5), CXC chemokine ligand 10 (CXCL10) and IL-10 were slightly downregulated without statistical significance (**Table 1** and **Figure 3**). The inflammatory state of the lungs can be directly represented by the cytokines in bronchoalveolar lavage fluid (BALF) (7, 31), we examined cytokines in BALF after 8 h of LPS stimulation. Twelve cytokines in total were simultaneously measured using a mouse inflammation panel by flow cytometric bead array. Compared with the model group, TNF-α and IL-6 were significantly decreased by the treatment of peramivir (**Table 2** and **Figures 2I, J**). Other cytokines such as IFN-γ, IFN-β, MCP-1, GM-CSF and so on had no significant difference by the treatment of peramivir compared with the model group (**Table 2** and **Figure 4**).

**Table 1:** Clinical feature and experimental results of cytokines/chemokines tested in mice serum.

Cytokines/chemokines	Elevated in COVID-19^3,43^	Higher in Severe Cases^3,43^	Control (pg/ml)	Peramivir (pg/ml)	*P*	Trend
TNF-α	Yes	Yes	1716.55 ± 82.10	1181.45 ± 47.58	<0.0001	↓
IL-6	Yes	No	101572.3 ± 5186.61	80316.8 ± 3464.71	0.0021	↓
IFN-α	No Data	No Data	649.52 ± 17.15	393.0 ± 16.59	<0.0001	↓
IFN-γ	Yes	No	1521.02 ± 154.27	516.95 ± 60.44	<0.0001	↓
IL-1β	Yes	No	578.21 ± 50.23	275.12 ± 28.97	0.0001	↓
IL-10	Yes	Yes	1048.84 ± 61.75	926.39 ± 66.67	0.25	−
IL-12	No	No	237.15 ± 7.88	119.17 ± 7.16	<0.0001	↓
CXCL1	No Data	No Data	31944.11 ± 6590.53	24037.25 ± 3011.44	0.44	−
IP10	Yes	Yes	44400.48 ± 2953.97	39370.0 ± 1970.93	0.27	−
MCP-1	Yes	Yes	35984.98 ± 1082.87	26563.21 ± 1257.91	<0.0001	↓
CCL-5	No Data	No Data	12179.03 ± 1014.57	9986.45 ± 455.71	0.19	−
GM-CSF	Yes	No	234.27 ± 12.01	190.45 ± 9.93	0.0157	↓

↓: Downward trend in the peramivir group vs control group; -: no significant trend in two groups.

**Table 2:** Clinical feature and experimental results of cytokines tested in mice BALF.

Cytokines	Elevated in COVID-19	Higher in Severe Cases	Control (pg/ml)	Peramivir (pg/ml)	*P*	Trend
TNF-α	No Data	No Data	26.39 ± 4.53	15.67 ± 2.56	0.03	↓
IL-6	No Data	No Data	10176.96 ± 2354.05	3916.37 ± 994.83	0.02	↓
IFN-β	No Data	No Data	30.92 ± 5.65	46.02 ± 9.69	0.23	−
IFN-γ	No Data	No Data	67.02 ± 20.29	100.06 ± 75.45	0.35	−
IL-1α	No Data	No Data	180.92 ± 50.29	186.23 ± 53.49	0.95	−
IL-1β	No Data	No Data	28.99 ± 5.99	22.21 ± 2.30	0.32	−
IL-10	No Data	No Data	43.11 ± 12.99	29.82 ± 7.20	0.38	−
IL-17A	No Data	No Data	26.81 ± 11.91	17.21 ± 4.12	0.47	−
IL-23	No Data	No Data	9.49 ± 1.87	11.45 ± 2.85	0.60	−
IL-27	No Data	No Data	22.16 ± 3.39	20.60 ± 4.25	0.79	−
MCP-1	No Data	No Data	900.08 ± 285.4	383.04 ± 71.79	0.12	−
GM-CSF	No Data	No Data	7.50 ± 1.38	10.08 ± 2.17	0.38	−

↓: Downward trend in the peramivir group vs control group; -: no significant trend in two groups.

### Peramivir Effectively Attenuates Acute Lung Injury and Prolongs the Survival in LPS-Induced Mice

**Figure 5:**
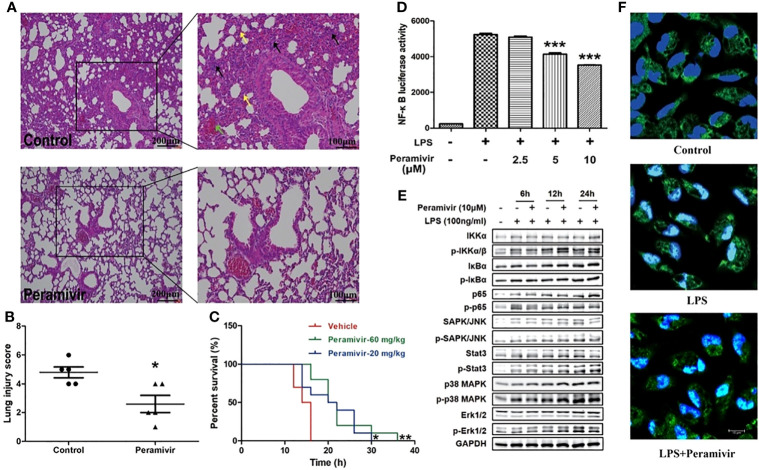
Peramivir effectively attenuates acute lung injury and prolong the survival in LPS-induced mice. **(A)** Representative images of lung H&E staining of control, and peramivir treatment groups. Black, green and yellow arrows indicated infiltration of inflammatory cells, congestion and edema within thickened alveolar, respectively. Scale bars, 100 or 200 μm as indicated. **(B)** Lung injury scores of control and peramivir treatment groups (n=5). *P < 0.05. **(C)** Survival time of LPS-induced CSS in control, and peramivir (20, 60 mg/kg) groups (n=10). Kaplan–Meier analysis was performed. *P < 0.05, **P < 0.01. **(D)** RAW264.7 cells were co-cultured with peramivir at concentrations of 2.5, 5 and 10 μM at 1 h before LPS stimulation. The activity of NF-κB luciferase was upregulated in all groups after 8 h, and there was a significant decline in cells co-cultured with peramivir in a dose-dependent manner. ***P < 0.001. **(E)** The activation of the NF-κB, MAPK and STAT pathway in LPS-stimulated macrophages after the treatment of peramivir. **(F)** p65 nuclear translocation in LPS-stimulated macrophages after the treatment of peramivir (blue, DAPI; green, p65; cyan, cyan). Scale bars, 10 μm as indicated.

The alveolar damage with cellular fibromyxoid exudates, pulmonary edema and interstitial mononuclear inflammatory infiltrates were both appeared in the histological examinations of two COVID-19 death cases (7, 31). The mice exhibited similar pathological features to ARDS, such as infiltration of inflammatory cells (black arrow), congestion (green arrow) and edema within thickened alveolar after the injection of LPS (yellow arrow) (**Figure 5A**). In contrast, the alveolar structures of mice in the peramivir treated group were relatively intact with less inflammatory cell infiltrations, mild alveolar thickening and less bleeding points or congestion were observed (**Figure 5A**). Peramivir showed significant protective effects (score = 2.6 ± 0.6) to the lung tissues compared with the control group (score = 4.8 ± 0.33) according to the Lung injury scores (**Figure 5B**). The survival time of mice was positively correlated with the concentration of peramivir injected into the intraperitoneal cavity after an i.p. injection of a lethal dose of LPS (30 mg/kg) compared with the control group (**Figure 5C**).

### Peramivir Decreases NF-κB Transcriptional Activity in RAW264.7 and the Peritoneal Macrophages

NF-κB is an important transcriptional regulator in cells that participated in inflammatory responses which can induce the expression of multiple genes and production of cytokines consequently leading to cytokine storm (32). Peramivir inhibited LPS-induced NF-κB transcriptional activity in the RAW264.7 cells with a NF-κB reporter luciferase system in a dose-dependent manner (**Figure 5D**). The LPS-induced activation of NF-κB pathway (phosphorylation of p65) or MAPKs (phosphorylation of p38 and Erk1/2) was inhibited after peramivir intervention (**Figure 5E**). Furthermore, immunofluorescence images showed that LPS-induced nuclear translocation of p65 was attenuated by peramivir (**Figure 5F**).

### Peramivir Inhibits Multi-Cytokine Releases in LPS-Induced hPBMCs

**Figure 6:**
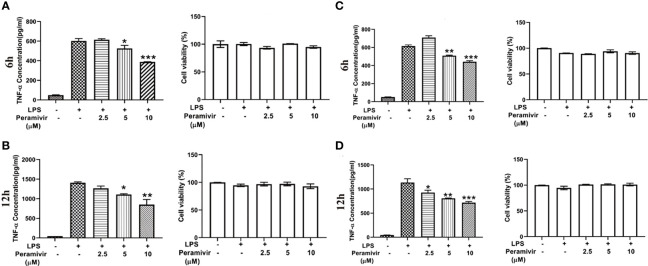
Peramivir inhibits cytokine release in LPS-induced hPBMCs from the two health donors. TNF-α concentration was elevated by LPS stimulation. Peramivir reduced TNF-α release at the time 6h **(A, C)** and 12h **(B, D)** with dose (2.5, 5, 10 μM)-dependent manner. Peramivir showed no toxicity toward hPBMCs. *P < 0.05, **P < 0.01, *** *P* < 0.001.

Considering the translational value of peramivir in clinical practices, the release of TNF-α was tested in LPS-induced hPBMCs, which were obtained from two healthy donors. Peramivir (**Figure 6**) significantly counteracted the level of TNF-α at 6 h and 12 h in a time- and dose-dependent manner without apparent toxicity.

## Discussion

The three neuraminidase inhibitors bear similar pharmacophoric side chains (black, **Figure 1A**) and different core scaffolds (red, **Figure 1A**). Peramivir has a five-membered cyclopentanol ring, while oseltamivir and zanamivir have six-membered ring, which might be a critical part for the anti-inflammatory activity in the chemical structure of view.

Peramivir is an intravenous neuraminidase inhibitor approved by the FDA in 2009 for emergency use in severe influenza. The antiviral effect of peramivir on influenza has been previously described (33), however, there are few reports on the anti-inflammatory activity of peramivir. Virus-induced cytokine responses are key to the activation of the immune system (34, 35). Inhibition of these cytokines can potentially control the severity of virus-induced inflammatory complications, ultimately reducing mortality (34, 35). Inflammatory cytokines and the pathogenicity of influenza (36, 37). In mouse model of H1N1 influenza, peramivir inhibits the levels of TNF-α, IL6 and IFN-γ in the lung tissue (38). And compared to oseltamivir, peramivir shows more obvious anti-inflammatory effect (39). The anti-inflammatory effect of peramivir *in vivo* may be due to its antiviral symptomatic treatment, and we sought to investigate whether peramivir could directly inhibit cytokine release from inflammatory immune cells.

As the neuraminidase is not expressed in the SARS-CoV-2 virus, oseltamivir, zanamivir and peramivir were ineffective against the virus *in vitro* (25). In a clinical study, 75% of the COVID-19 patients received antiviral treatment including oseltamivir and 5 of them simultaneously infected with SARS-CoV-2 and influenza were recovered after treating with oseltamivir (23). We hypothesized that these neuraminidase inhibitors might have other effects including anti-inflammation, indicating the adjuvant therapeutic value of neuraminidase inhibitors in COIVD-19.

Viruses and bacteria induce immune cell activation and release of cytokines are Toll-like receptors (TLRs) dependent (40). The induction of inflammatory cytokines depends on the activation of NF-κB although they recognize different subtypes of TLRs (41). We assumed that SARS-CoV-2 may activate NF-κB on cytokine storm similar to that of SARS-CoV (41). LPS activates immune cells such as monocytes and macrophages, causing the synthesis and release of inflammatory cytokines (28). TNF-α is one of the central cytokines involved in inflammation initiation and amplification in virus infections (42), and is reported to be elevated in critical COVID-19 cases (3, 43), suggesting it as a proper indicator for *in vitro* drug screening.

Compared with oseltamivir, peramivir is more effective in inhibiting TNF-α *in vitro*. The reduction of TNF-α, IL-1β, IL-6, IL-12, IFN-α, IFN-γ and MCP-1 levels promoted by peramivir may play an important role in reducing stress and prevention caused by excessive activation of the immune system. This hypothesis was confirmed by pathological examination and lung index evaluation, and peramivir treatment was found to reduce the severity of LPS-associated pneumonia and prolong the survival time of mice. The lethal lung pathology caused by LPS is due to an excessive cytokine response produced primarily by activated macrophages (28). Peramivir inhibit that levels of TNF-α and IL-6 in BALF of mice induce by LPS. Furthermore, peramivir attenuated LPS-induced TNF-α in mouse peritoneal macrophages and hPBMCs, confirming that peramivir can inhibit inflammatory cytokine responses mediated by macrophages.

In conclusion, the regulatory function of peramivir on LPS-induced inflammatory cytokines may contribute to the additional beneficial effect of this drug in antiviral therapy. The study provides evidence of therapeutic value for the potential use of peramivir as an anti-inflammatory agent against cytokine dysregulation.

## Data Availability Statement

The original contributions presented in the study are included in the article/supplementary material. Further inquiries can be directed to the corresponding authors.

## Ethics Statement

All animal experiments were carried out in adherence with the NIH Guide for the Care and Use of Laboratory Animals (National Academies Press, 2011) and were reviewed and approved by the Second Military Medical University Committee on Animal Care (EC11-055).

## Author Contributions

LS, C-LZ, W-NZ, and Z-BW conceived and designed the experiments. LS, YT, X-CS, D-GC, C-XZ, and Z-BW participated in the experiments; LS, YT, and Z-BW analyzed the data. LS, C-LZ, X-CS, and Z-BW wrote the manuscript. All the authors provided the final approval of the manuscript.

## Funding

This work was supported in part by grants from the National Natural Science Foundation of China (81872880, 81703506) and the Young Elite Scientists Sponsorship Program by the China Association for Science and Technology (2017QNRC061).

## Conflict of Interest

The authors declare that the research was conducted in the absence of any commercial or financial relationships that could be construed as a potential conflict of interest.

## Publisher’s Note

All claims expressed in this article are solely those of the authors and do not necessarily represent those of their affiliated organizations, or those of the publisher, the editors and the reviewers. Any product that may be evaluated in this article, or claim that may be made by its manufacturer, is not guaranteed or endorsed by the publisher.
